# Investigation of bioactivities of *Taxus chinensis*, *Taxus cuspidata*, and *Taxus × media* by gas chromatography-mass spectrometry

**DOI:** 10.1515/biol-2021-0032

**Published:** 2021-03-23

**Authors:** Shuqiang Zhang, Xueyan Lu, Tianyao Zheng, Xiaorui Guo, Qi Chen, Zhonghua Tang

**Affiliations:** School of Life Sciences, Nantong University, Nantong 226010, China; Key Laboratory of Plant Ecology, Northeast Forestry University, Harbin 150040, China

**Keywords:** *Taxus chinensis*, *Taxus cuspidata*, *Taxus × media*, bioactivities, gas chromatography-mass spectrometry

## Abstract

*Taxus* species have attracted much attention for their potency in cancer treatment. However, investigating the bioactivities of *Taxus* species is a complex task, due to their diversity, slow growth, and endangered state. The most important *Taxus* species in China are *Taxus chinensis* (*T. chinensis*), *Taxus cuspidata* (*T. cuspidata*), and *Taxus* × *media* (*T. media*), which mainly grow in the northeastern region. This article probes deep into the differences among the leaves of *T. chinensis*, *T. cuspidata*, and *T. media*, with the aid of gas chromatography-mass spectrometry (GC-MS). Through GC-MS, 162 compounds were detected in the samples and found to contain 35 bioactive metabolites. On this basis, 20 metabolites with significant bioactivities (antibiotic, antioxidant, anticancer, and antiaging effects) were identified via unsupervised learning of principal component analysis and supervised learning of partial least squares-discriminant analysis. The results show that *T. media* has the most prominent antibiotic, antioxidant, and anticancer effects, while *T. cuspidata* has the most diverse and abundant metabolites that slow down aging.

## 1 Introduction

*Taxus* species are rich in a natural antitumor substance called taxol [1]. This substance can keep tubulin stable and inhibit cell mitosis, exhibiting a strong radio-sensitizing effect [2]. There is a good evidence that taxol is a potent drug against various cancers. As a result, taxol has been applied alone or with other anticancer agents to treat breast, ovarian, and lung cancers [2,3]. To date, more than 400 taxoids and modified taxoids have been isolated for characterization from the bark, seeds, and leaves of the genus *Taxus* [4,5].

Each *Taxus* species has its unique properties and, thus, a particular way of use. Most plants in the Taxus family are endangered evergreen trees or shrubs that grow rather slowly [1,5]. Of the various *Taxus* species, three are mainly used in northeastern China, namely, *Taxus chinensis* (*T. chinensis*, N), *Taxus cuspidata* (*T. cuspidata*, D), and *Taxus × media* (*T. media*, M). *T. chinensis* is an evergreen conifer native to China, where it is also referred to as “Beauteous Taxus” [6]. It grows across southeastern China, including Jiangxi, Fujian, Hunan, and Taiwan [5]. Being an endemic plant to China, *T. chinensis* is under protection for its high value as a natural anticancer plant [7]. The extracts of the plant have been commonly used in traditional Chinese medicine for cancer treatment [7,8]. *T. cuspidata* is a low-trailing and evergreen tree or shrub and the most extensively studied *Taxus* species. This plant mainly grows in the northeastern mountains of China, the Korean Peninsula, and Japan. It is also a very popular ornamental tree in Japan and North America. Apart from being a garden tree, *T. cuspidata* has been used as a crude drug to treat diabetes, promote diuresis, and stimulate menstrual flow [9]. More than 120 new taxoids have been discovered in *T. cuspidata* [3]. *T. media* is an evergreen shrub with a huge biomass, a high growth rate, and a strong adaptability to the environment [10]. It is the natural hybrid from *T. cuspidata*, the female parent, and *Taxus baccata* (*T. baccata*), the male parent [10]. The plant has been growing for almost a century in North America [10]. *T. media* boasts a lush foliage, a strong resistance to cold, and a high ornamental value. Overall, the *Taxus* species are well-known for their precious medicinal and greening effects. Currently, the research mainly focuses on the taxol in different *Taxus* species. For example, the transcriptome analyses provide insights into the expression pattern and sequence similarity of several taxol biosynthesis-related genes [11]. And the comparative metabolomic analysis reveals the variations in taxoids and flavonoids among the *Taxus* species [12]. However, there is not yet a clear comparison of bioactivities among *T. chinensis*, *T. cuspidata*, and *T. media* in bioactivities [13]. The bioactivities include antibiotic, antioxidant, anticancer, and antiaging effects in medical plants [14]. To make up for the gap, this article compares the difference among the leaves of *T. chinensis*, *T. cuspidata*, and *T. media*, with the aid of gas chromatography-mass spectrometry (GC-MS).

The GC-MS is the most popular technique used to identify and quantify plant metabolomics. Metabolomics approaches have been in the spotlight as a powerful tool to gain comprehensive information of the metabolic network and to significantly identify the different metabolites related to the defense mechanisms [15,16,17]. And GC-MS analysis predominantly focuses on the identification and quantification of small polar and volatile components, e.g., primary metabolites such as amino acids, sugars, and organic acids. Through GC-MS, 162 compounds were detected in the samples and found to contain 35 bioactive metabolites. Then 20 significant metabolites were identified via principal component analysis (PCA) and partial least squares-discriminant analysis (PLS-DA) and divided into four classes based on bioactivity. The results show that *T. media* has the most prominent antibiotic, antioxidant, and anticancer effects, and *T. cuspidata* is the most suitable choice to slow down aging.

## 2 Materials and methods

### 2.1 Materials

*T. chinensis*, *T. cuspidata*, and *T. media* were grown in a growth chamber at the temperature of 25°C (day)/18°C (night) and the relative humidity of 45%. All necessary nutrients were supplied to ensure the normal growth of the plants. Before flowering, the well-grown leaves of three species were selected and divided into three groups, each of which contains six repeated samples.

### 2.2 Sample preparation

Leave tissues of 60 mg were weighted and mixed with 360 μL of cold methanol and 40 μL of internal standards (0.3 mg/mL 2-chlorophenylalanine in methanol). The mixture was homogenized by a Tissuelyser-192 (Jingxin, Shanghai). After 30 min of ultrasonication, the sample was added with 200 μL of chloroform and 400 μL of water. The mixture was vortexed for 2 min and sonicated for 30 min, before being centrifuged at 10,000 *g* for 10 min at 4°C. Next 400 μL of supernatant was relocated to a glass sample vial and vacuum dried at room temperature. The residue was derivatized in two steps: first, 80 μL of methoxyamine (15 mg/mL in pyridine) was added to the vial and the mixture was vortexed for 30 s and kept at 37°C for 90 min; second, 80 μL of *N,O*-bis(trimethylsilyl)trifluoroacetamide, 1% trimethylchlorosilane, and 20 μL of *n*-hexane were added and the mixture was kept at 70°C for 60 min.

### 2.3 GC-MS analysis

Each 1 μL of aliquot of the derivatized solution was injected into an Agilent 7890A-5975C GC-MS system (Agilent, USA) with a split ratio of 30:1. Separation was carried out on a nonpolar Agilent J&W DB-5 capillary column (inner diameter: 30 m × 250 μm; Agilent, USA), with high-purity helium as the carrier gas at a constant flow of 1.0 mL/min.

The GC temperature was programmed as follows: the initial oven temperature was set to 50°C, which was increased at 8°C/min to 125°C, 15°C/min to 170°C, 4°C/min to 210°C, 10°C/min to 270°C, and 5°C/min to 305°C; the final temperature of 305°C was maintained for 5 min. The temperature of injection and ion source was set to 260°C and 230°C, respectively.

Electron ionization (−70 eV) at full scan mode (*m/z* 30–600) was used, with an acquisition rate of 20 spectrum/s in the MS setting. Throughout the analytical run, the quality control (QC) samples were injected at regular intervals (every 10 samples) to provide a set of data with assessable repeatability. Each QC sample was prepared by mixing aliquots of the tissue samples into a pooled sample and then analyzed by the same method as the analytic samples.

### 2.4 Data extraction and analysis

The MS data acquired through GC-MS were analyzed by ChromaTOF (v 4.34; LECO, USA). After being aligned with Statistic Compare component, the comma separated vector file was obtained with three-dimensional (3D) data sets, including sample information, retention time, and peak intensities. Then each data set was normalized using the total peak intensity of each sample.

The data sets obtained by GC-MS were separately imported into SIMCA 13.0 (Umetrics, Sweden). Then PCA and PLS-DA were carried out to visualize the metabolic alterations among the test groups, after mean centering and unit-variance scaling. The default sevenfold cross validation was applied. To prevent overfitting, 1/7 of the samples were excluded from the model in each round.

During the experiments, all differentially expressed compounds were selected by comparing the compounds among *T. chinensis*, *T. cuspidata*, and *T. media* through multivariate statistical analysis, Student’s *t* test, and Mann–Whitney *U* test. The metabolites with both multivariate and univariate statistical significance (variable importance in projection [VIP] > 1.0 and *p* < 0.05) were identified. The similarity of more than 70% was considered as the reference standard. According to the PLS-DA results, the overall contribution of each variable to the PLS-DA model was ranked by VIP, and the variables with VIP > 1.0 were deemed as relevant for group discrimination.

### 2.5 Statistical analysis

To improve normality, the metabolite data were log_2_ transformed and normalized. A total of 18 samples were subject to clustering analysis by *R*, revealing the variations in *T. chinensis*, *T. cuspidata*, and *T. media* in leaf tissues. Then a heat map and a box plot were drawn to display the structure of the experimental data with R-3.2.0 and SigmaPlot 10.0, respectively.

## 3 Results and discussion

**Figure 1:**
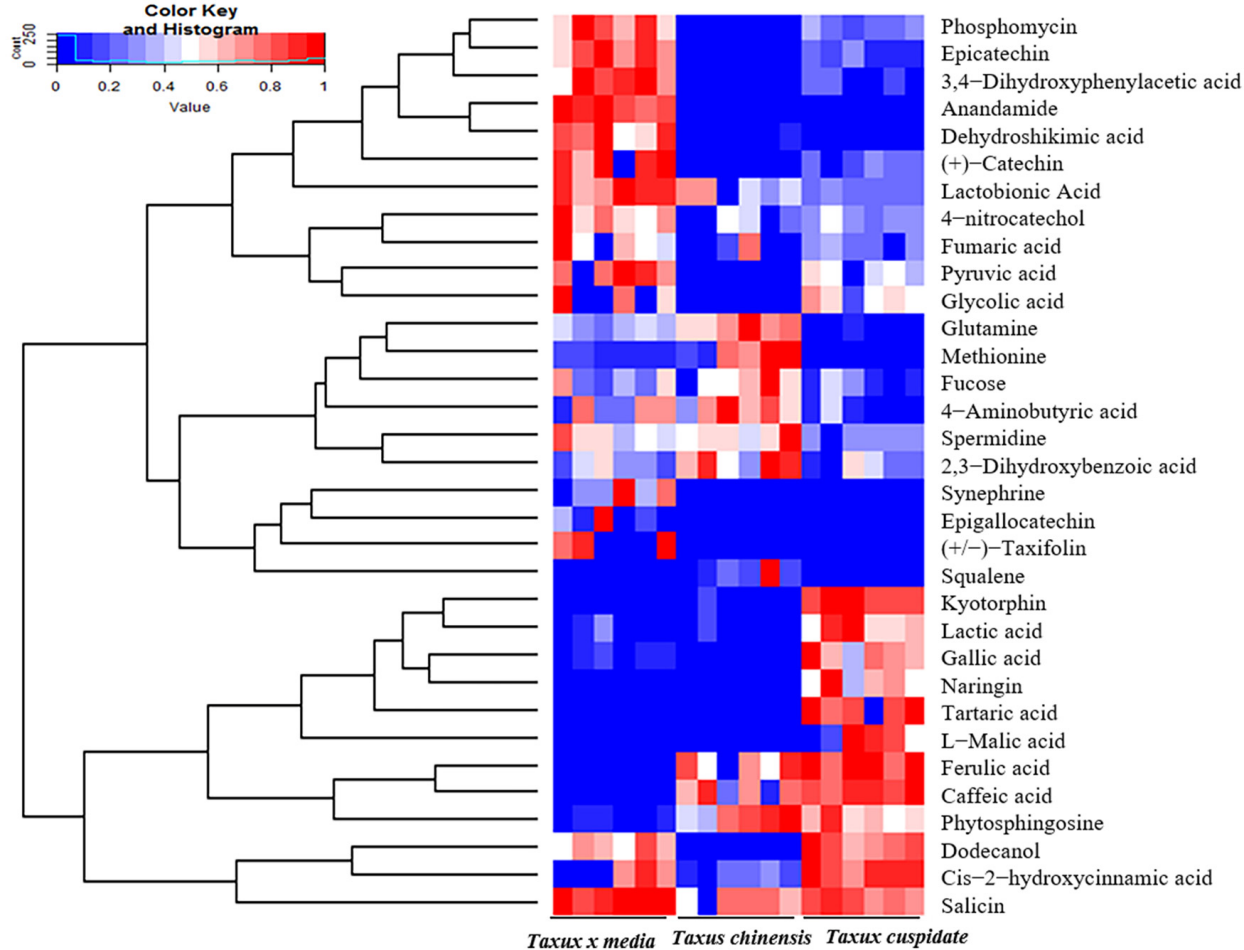
The heat map on the relative differences of compounds among *T. chinensis*, *T. cuspidata*, and *T. media*. Note: The relative content of each metabolite was normalized to complete the hierarchical clustering; high and low abundances are in red and blue, respectively.

*Taxus* species is a widely favored landscape plant [18]. But only a few studies have compared the bioactivities of different *Taxus* species. In this article, the GC-MS is performed to detect the differences among the leaves of *T. chinensis*, *T. cuspidata*, and *T. media*. A total of 162 compounds were obtained from the GC-MS analysis. And 35 metabolites were selected based on antibiosis, antioxidant, anticancer, and antiaging effects. The content value of each metabolite was normalized to complete the linkage hierarchical clustering. Then the heat map visualization of relative differences of metabolites among *T. chinensis*, *T. cuspidata*, and *T. media* is shown in Figure 1. The metabolites were phosphomycin, epicatechin, 3,4-dihydroxyphenylacetic acid, anandamide, dehydroshikimic acid, catechin, lactobionic acid, 4-nitrocatechol, fumaric acid, pyruvic acid, glycolic acid, glutamine, methionine, fucose,4-aminobutyric acid, spermidine, 2,3-dihydroxybenzoic acid, synephrine, epigallocatechin, taxifolin, squalene, kyotorphin, lactic acid, gallic acid, naringin, tartaric acid, l-malic acid, ferulic acid, caffeic acid, phytosphingosine, dodecanol, *cis*-2-hydroxycinnamic acid, salicin, from top to bottom, in turn.

**Figure 2:**
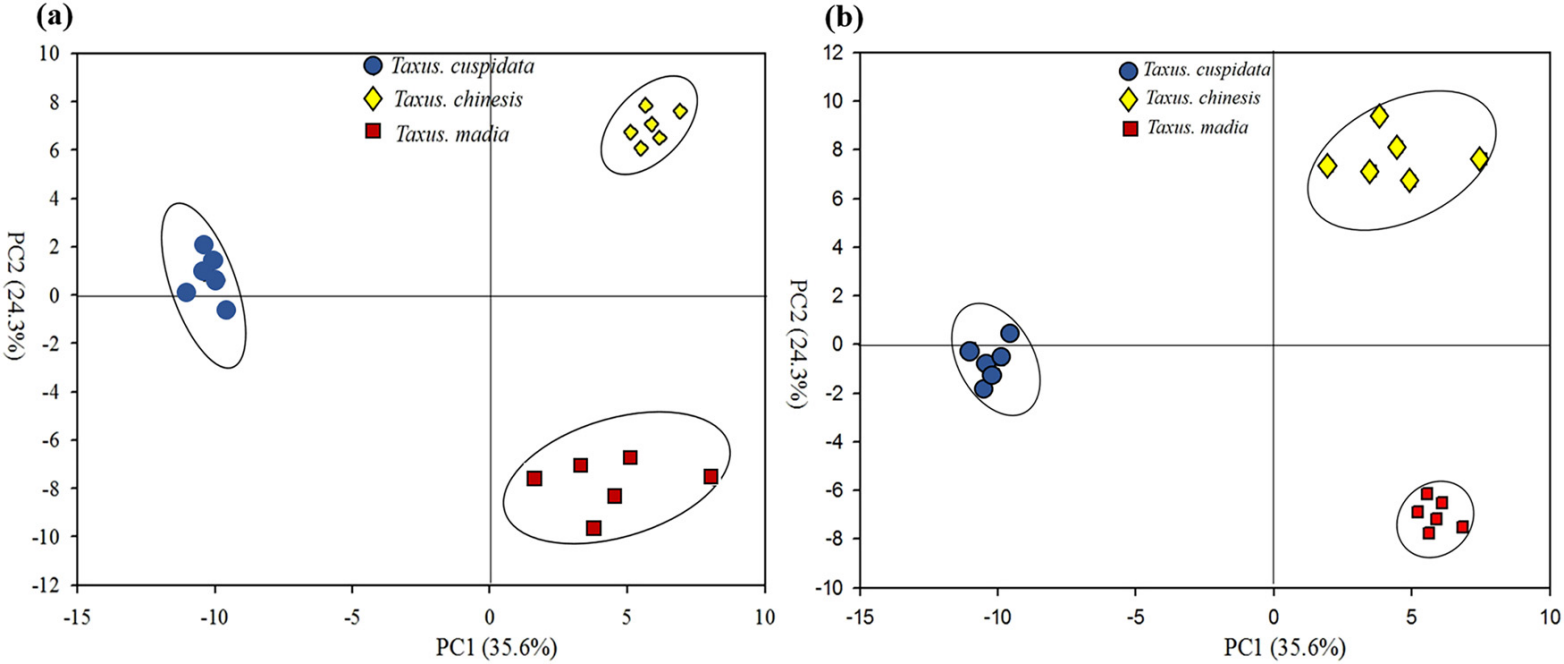
The PCA score plot (a) and PLS-DA score plot (b) of compounds among *T. chinensis*, *T. cuspidata*, and *T. media.* Note: Blue circle, yellow diamond, and red square represent *T. chinensis*, *T. cuspidata*, and *T. media*, respectively.

To identify the significant metabolites, the dimensionality of the GC-MS data was reduced; and the grouping of samples was visualized through the PCA, an unsupervised multivariate analysis technique, and PLS-DA, a supervised multivariate analysis technique. In the PCA score plot, three species had significant separations, whose interpretability and predictability were 35.6 and 24.3%, respectively (Figure 2a).

As shown in Table 1, from the PLS-DA of three species, a total of 85 significant metabolites were selected under the criteria of VIP > 1 and *p* < 0.05. These metabolites differed sharply in abundance within the leaf tissues of different species and contributed greatly to leaf discrimination between three species. Then the metabolites were classified into 16 amino acids, 11 sugars, 28 acids, 6 alcohols, 7 amines, and 6 unclassified compounds. Among them, amino acids and amines were mainly detected in *T. cuspidata* (12 and 5, respectively), while sugars, acids, and alcohols were mainly detected in *T. media* (6, 14, and 3, respectively).

**Table 1:** Different metabolites among *T. chinensis*, *T. cuspidata*, and *T. media*

	Metabolites	VIP	Relative content
Amino acids	Citrulline	1.26	M > D
	Methionine	1.05	D
	*N*-Epsilon-acetyl-l-lysine	1.04	D
	Norleucine	1.04	D
	3-Hydroxynorvaline	1.01	D
	Lysine	1.19	D > M > N
	Threonine	1.19	D > M > N
	Alanine	1.18	D > M > N
	Proline	1.18	D > M > N
	Phenylalanine	1.17	N > M > D
	Isoleucine	1.16	D > M > N
	Nicotinoylglycine	1.16	N > M > D
	Beta-alanine	1.16	D > N > M
	Serine	1.12	D > M > N
	Ornithine	1.10	M > D
	Valine	1.10	D > M > N
Sugars	Melezitose	1.34	N > M > D
	Sedoheptulose	1.33	M > D
	Trehalose	1.19	N > M
	Salicin	1.18	M > D
	Prunin	1.35	N
	Levoglucosan	1.16	N > M > D
	Lactose	1.15	M > D
	Isopropyl-beta-d-thiogalactopyranoside	1.11	M > D > N
	Fucose	1.11	D
	Maltotriitol	1.11085	M > D > N
	Glucose	1.05	M > D > N
Acids	Pelargonic acid	1.34	M
	3,4-Dihydroxyphenylacetic acid	1.33	M > D
	2-Hydroxybutanoic acid	1.33	M > D > N
	*o*-Hydroxyhippuric acid	1.32	M > D
	Dehydroshikimic acid	1.31	M > N
	2-Methylglutaric acid	1.30	M
	Hippuric acid	1.32	M > D
	Fumaric acid	1.23	N > D > M
	l-Malic acid	1.23	N > M > D
	Aminooxyacetic acid	1.20	N > M > D
	3-Hydroxypropionic acid	1.19	D
	2,3-Dihydroxybenzoic acid	1.19	D > N > M
	Pipecolinic acid	1.18	D
	Itaconic acid	1.18	N > M > D
	2-Hydroxy-3-isopropylbutanedioic acid	1.18	D > M
	Glycolic acid	1.16	D > N
	Caffeic acid	1.15	M > D
	Pyruvic acid	1.15	D > N > M
	4-Aminobutyric acid	1.13	D > M > N
	Oxalic acid	1.11	N > D > M
	*cis*-2-Hydroxycinnamic acid	1.10	M > N > D
	Galactonic acid	1.08	M > D > N
	Threonic acid	1.08	M > D > N
	Quinic acid	1.06	M > D > N
	d-Glyceric acid	1.05	D > M > N
	Tartaric acid	1.05	M > D > N
	Allylmalonic acid	1.04	M > D
	6-Hydroxy caproic acid	1.02	D
Amines	Anandamide	1.37	M > D > N
	Ethanolamine	1.19	D > M > N
	*N*-Omega-acetylhistamine	1.17	D
	5-Methoxytryptamine	1.14	D > N
	Methoxamedrine	1.13	N > M > D
	Lactamide	1.12	D
	Spermidine	1.11	D > M > N
Alcohols	Acetol	1.36	N
	2-Amino-1-phenylethanol	1.18	D > M > N
	Allo-inositol	1.18	M > N > D
	2-Aminoethanethiol	1.16	N > M > D
	Dodecanol	1.12	M > D > N
	Myo-inositol	1.01	M > N > D
Others	Phosphomycin	1.36	M > D
	4-Nitrocatechol	1.31	D > M > N
	4-Androsten-11 beta-ol-3,17-dione	1.08	D > M > N
	1,3-Diaminopropane	1.07	D > M > N
	4-Hydroxybutyrate	1.03	M > N > D
	Squalene	1.02	M

Note: VIP is the variable importance in projection (VIP > 1; *p* < 0.05); N, D, and M represent *T. chinensis*, *T. cuspidata*, and *T. media*, respectively.

From Figures 1 and 2, 20 metabolites with different significant bioactivities (*p* < 0.05) were identified, including anandamide, phosphomycin, epicatechin, 3,4-dihydroxyphenylacetic acid, dehydroshikimic acid, fumaric acid, l-malic acid, 2,3-dihydroxybenzoic acid, salicin, caffeic acid, pyruvic acid, 4-aminobutyric acid, dodecanol, spermidine, fucose, *cis*-2-hydroxycinnamic acid, tartaric acid, methionine, 4-hydroxybenzoic acid, and squalene. The identified metabolites were classified by four kinds of bioactivities, namely, antibiotic effect, antioxidant effect, anticancer effect, and antiaging effect (Table 2).

**Table 2:** Four classes of significant metabolites among *T. chinensis*, *T. cuspidata*, and *T. media*

Bioactivities	Metabolites	Relative content
Antibiotic effect	Phosphomycin	M > D
	Fumaric acid	N > D > M
	Dodecanol	M > D > N
	*cis*-2-Hydroxycinnamic acid	M > N > D
	Caffeic acid	M > D
	4-Hydroxybenzoic acid	M > N > D
	3,4-Dihydroxyphenylacetic acid	M > D
Antioxidant effect	Caffeic acid	M > D
	4-Hydroxybenzoic acid	M > N > D
	3,4-Dihydroxyphenylacetic acid	M > D
	l-Malic acid	N > M > D
	Tartaric acid	M > D > N
	Pyruvic acid	D > N > M
	Epicatechin	M > D > N
	Anandamide	M > D > N
	Dehydroshikimic acid	M > N > D
Anticancer effect	2,3-Dihydroxybenzoic acid	D > N > M
	Caffeic acid	M > D
	4-Hydroxybenzoic acid	M > N > D
	Salicin	M > D
	Fucose	D
	Squalene	M
	3,4-Dihydroxyphenylacetic acid	M > D
	Anandamide	M > D > N
	Dehydroshikimic acid	M > N > D
Anti-aging effect	4-Aminobutyric acid	D > M > N
	Spermidine	D > M > N
	Squalene	M
	Methionine	D

Note: N, D, and M represent *T. chinensis*, *T. cuspidata*, and *T. media*, respectively.

**Figure 3:**
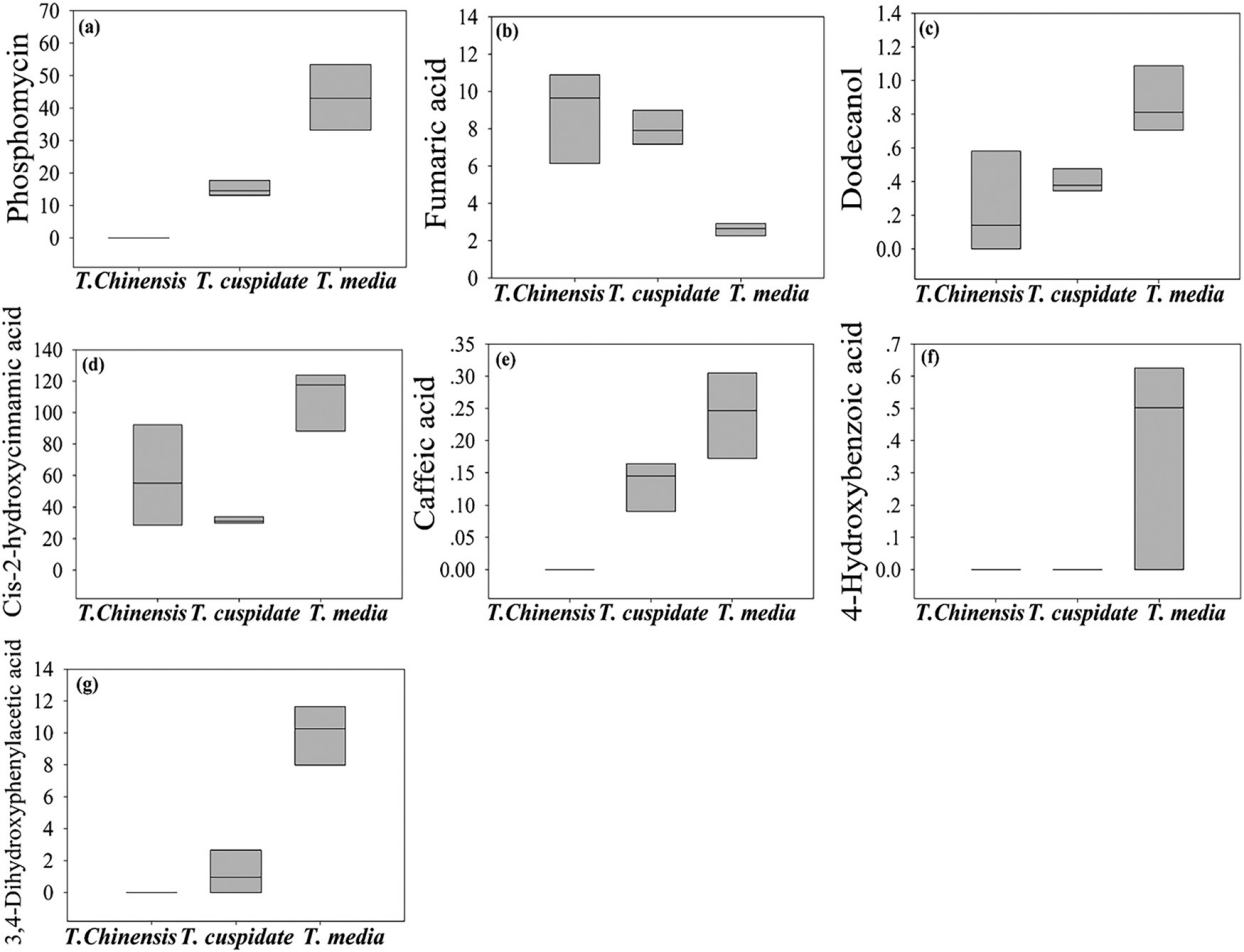
The content of metabolites with significant antibiotic effect. (a) Phosphomycin, (b) fumaric acid, (c) caffeic acid, (d) *cis*-2-hydroxycinnamic acid, (e) 4-hydroxybenzoic acid, (f) dodecanol, and (g) 3,4-dihydroxyphenylacetic acid.

As shown in Table 2 and Figure 3, seven metabolites were found to have significant antibiotic effect. These metabolites were mainly detected in the leaves of *T. cuspidata* and *T. media*. The highest contents of these metabolites were observed in *T. media*. Among them, dodecanol has an antifungal effect [19]. Phosphomycin, fumaric acid, caffeic acid, *cis*-2-hydroxycinnamic acid, 4-hydroxybenzoic acid, and 3,4-dihydroxyphenylacetic acid boast the broad-spectrum antibacterial property. Phosphomycin, 4-hydroxybenzoic acid, and 3,4-dihydroxyphenylacetic acid can inhibit the synthesis of cellular structure [20,21,22]. Fumaric acid, *cis*-2-hydroxycinnamic acid, and caffeic acid can regulate the osmotic pressure of cells [23,24]. By antibiotic effect, three *Taxus* species can be ranked in the descending order as *T. media*, *T. cuspidata*, and *T. chinensis*.

**Figure 4:**
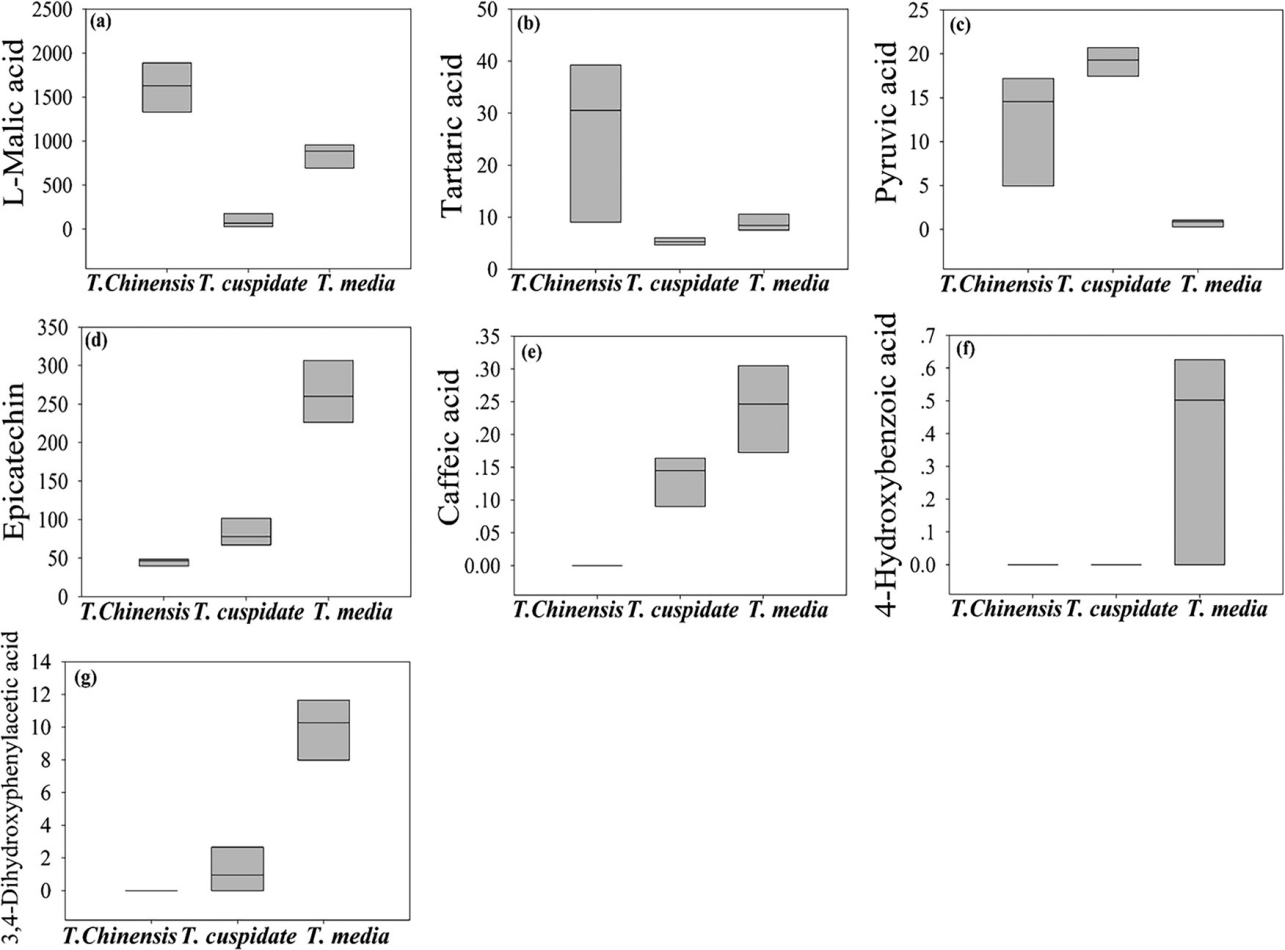
The content of metabolites with significant antioxidant effect. (a) l-malic acid, (b) tartaric acid, (c) pyruvic acid, (d) epicatechin, (e) caffeic acid, (f) 4-hydroxybenzoic acid, and (g) 3,4-dihydroxyphenylacetic acid.

As shown in Table 2 and Figure 4, seven metabolites were found to have significant antioxidant effects. Among them, caffeic acid, 4-hydroxybenzoic acid, 3,4-dihydroxyphenylacetic acid, tartaric acid, and epicatechin were prominently accumulated in *T. media*; l-malic acid was mainly accumulated in *T. cuspidata*; and pyruvic acid was mainly accumulated in *T. chinensis*. These metabolites can effectively suppress the oxidation of free radicals by directly interfering in or indirectly consuming these radicals [25,26,27]. By antioxidant effect, three *Taxus* species can be ranked in the descending order as *T. media*, *T. cuspidata*, and *T. chinensis*.

**Figure 5:**
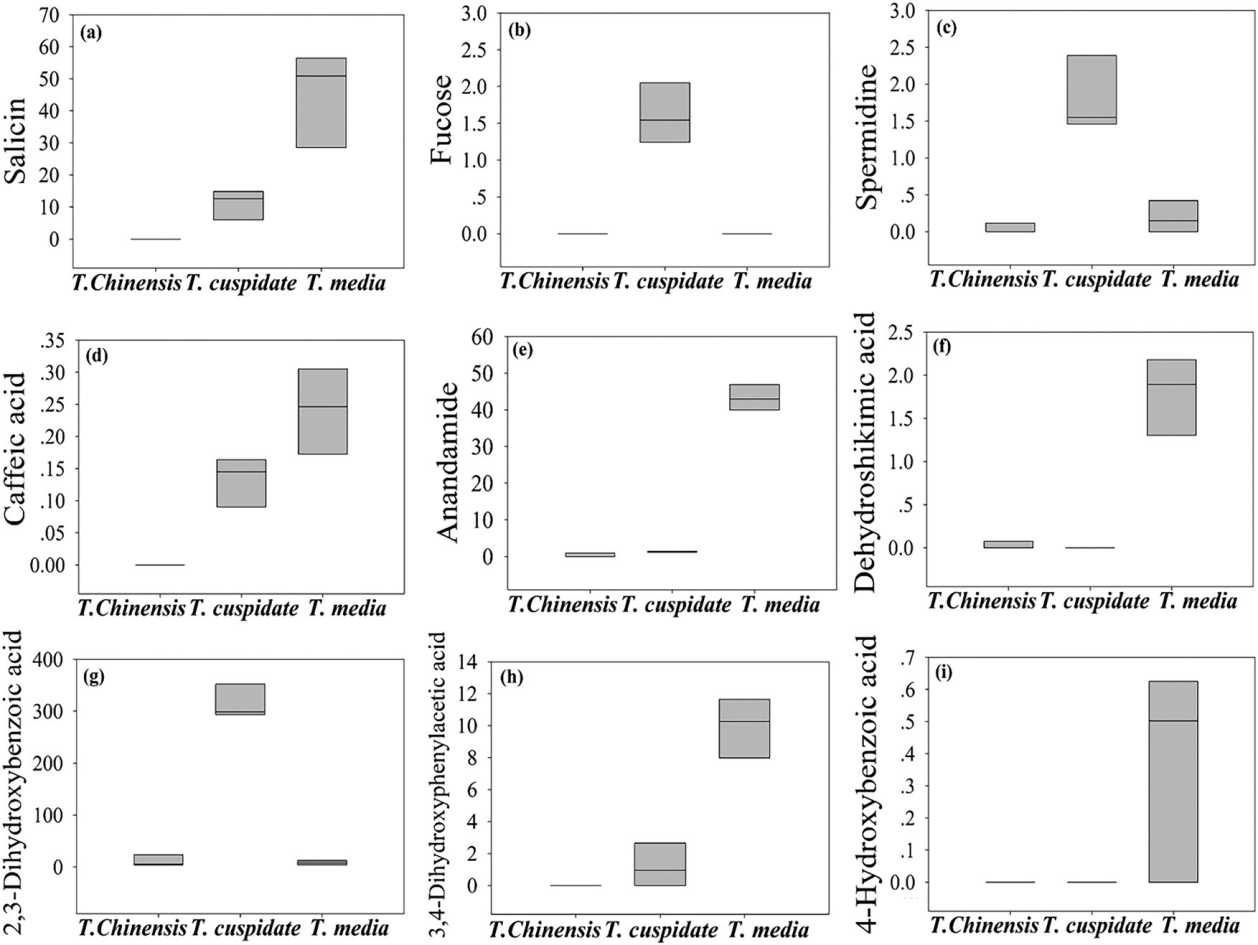
The content of metabolites with significant anticancer effect. (a) Salicin, (b) fucose, (c) squalene, (d) caffeic acid, (e) anandamide, (f) dehydroshikimic acid, (g) 2,3-dihydroxybenzoic acid, (h) 3,4-dihydroxyphenylacetic acid, and (i) 4-hydroxybenzoic acid.

As shown in Table 2 and Figure 5, nine metabolites were found to have significant anticancer effects. Among them, caffeic acid, 4-hydroxybenzoic acid, 3,4-dihydroxyphenylacetic acid, squalene, anandamide, dehydroshikimic acid, and salicin were observably accumulated in *T. media*; only 2,3-dihydroxybenzoic acid and fucose were dramatically accumulated in *T. cuspidata*. Their anticancer effect mainly comes from the interference in normal metabolism of cancer cells or the destruction of the cell structure [20,21,27,28,29,30]. The best anticancer effect was observed in *T. media*.

**Figure 6:**
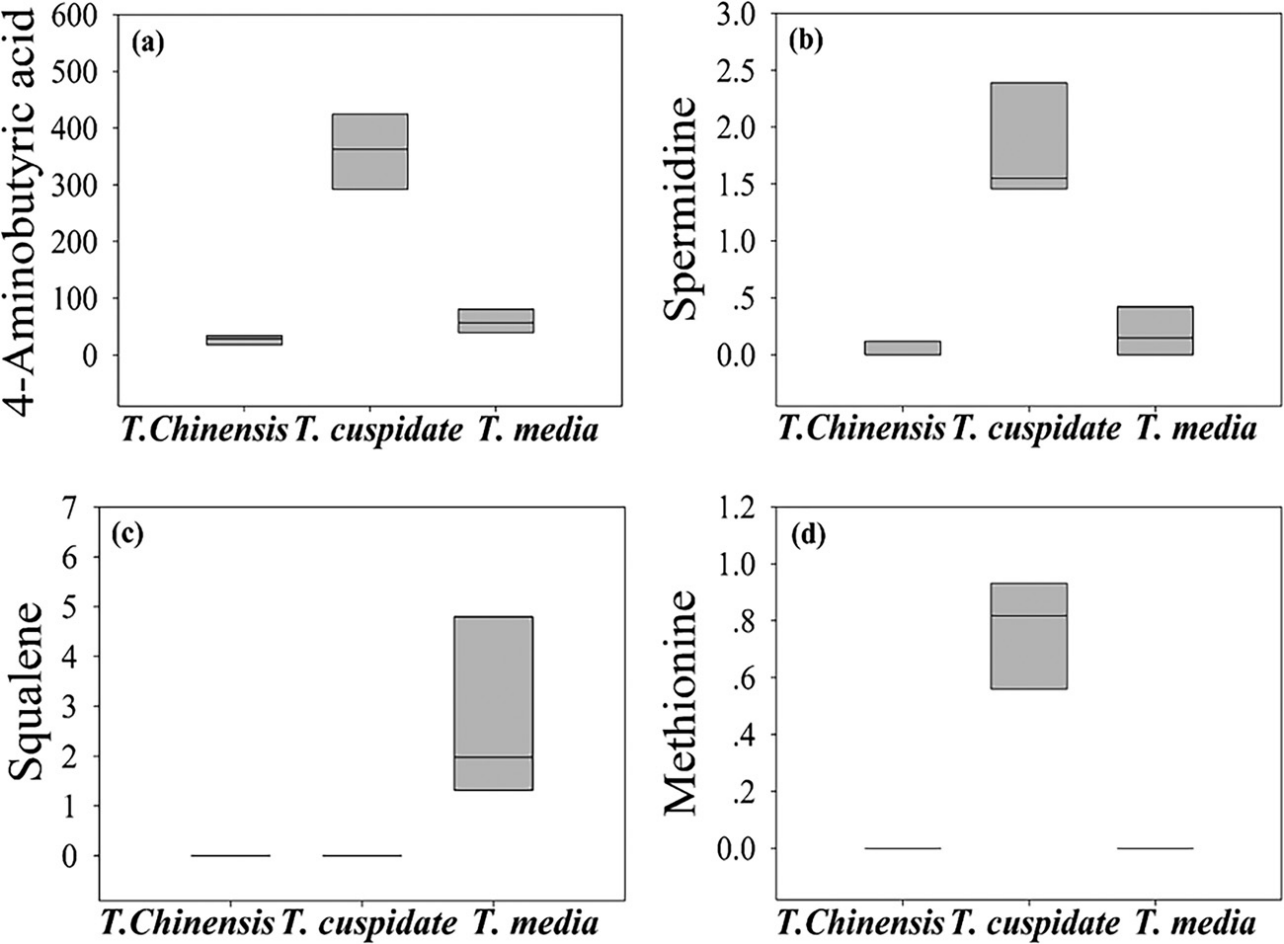
The content of metabolites with significant antiaging effect. (a) 4-Aminobutyric acid, (b) spermidine, (c) squalene, and (d) methionine.

As shown in Table 2 and Figure 6, four metabolites were found to have significant antiaging effects: 4-aminobutyric acid, spermidine, squalene, and methionine. Specifically, methionine, which was only detected in *T. cuspidata*, can affect the key physiologically active substances of antidepressant [31]. Both 4-aminobutyric acid and spermidine mainly existed in *T. cuspidata*. The former is an inhibitory neurotransmitter capable of activating the brain and delaying brain aging, while the latter delays protein aging by inhibiting the synthase of neuronal nitric oxide and mitigates age-related memory loss [32]. Squalene, which was detected in *T. media*, exerts biological redox effect and improves the energy efficiency, thereby enhancing immunity and slowing down aging [33]. By antiaging effect, the three *Taxus* species can be ranked in the descending order as *T. cuspidata*, *T. media*, and *T. chinensis*. To sum up, *T. media* is an excellent ornamental plant with excellent antibiotic, antioxidant, and anticancer effects; *T. cuspidata* stands out in antiaging effect.

## 4 Conclusions

This article mainly compares the levels and bioactivities of metabolites in the leaves of *T. chinensis*, *T. cuspidata*, and *T. media* through untargeted metabolomics GC-MS. The differences in bioactivity were found to vary with the relative contents of these metabolites in three species. The results show that *T. media* has the most prominent antibiotic, antioxidant, and anticancer effects, while *T. cuspidata* is the most suitable choice to slow down aging. The research results provide a reference for improving human health with *Taxus* species and for applying GC-MS to other ornamental plants.
